# Instances of erroneous DNA barcoding of metazoan invertebrates: Are universal *cox1* gene primers too “universal”?

**DOI:** 10.1371/journal.pone.0199609

**Published:** 2018-06-22

**Authors:** Monika Mioduchowska, Michał Jan Czyż, Bartłomiej Gołdyn, Jarosław Kur, Jerzy Sell

**Affiliations:** 1 Department of Genetics and Biosystematics, Faculty of Biology, University of Gdansk, Gdansk, Poland; 2 Research Centre of Quarantine, Invasive and Genetically Modified Organisms, Institute of Plant Protection – National Research Institute, Poznań, Poland; 3 Department of General Zoology, Institute of Environmental Biology, Faculty of Biology, Adam Mickiewicz University, Poznań, Poland; 4 Institute of Nature Conservation, Polish Academy of Science, Kraków, Poland; University of Guelph, CANADA

## Abstract

The cytochrome c oxidase subunit I (*cox1*) gene is the main mitochondrial molecular marker playing a pivotal role in phylogenetic research and is a crucial barcode sequence. Folmer’s “universal” primers designed to amplify this gene in metazoan invertebrates allowed quick and easy barcode and phylogenetic analysis. On the other hand, the increase in the number of studies on barcoding leads to more frequent publishing of incorrect sequences, due to amplification of non-target taxa, and insufficient analysis of the obtained sequences. Consequently, some sequences deposited in genetic databases are incorrectly described as obtained from invertebrates, while being in fact bacterial sequences. In our study, in which we used Folmer’s primers to amplify COI sequences of the crustacean fairy shrimp *Branchipus schaefferi* (Fischer 1834), we also obtained COI sequences of microbial contaminants from *Aeromonas* sp. However, when we searched the GenBank database for sequences closely matching these contaminations we found entries described as representatives of Gastrotricha and Mollusca. When these entries were compared with other sequences bearing the same names in the database, the genetic distance between the incorrect and correct sequences amplified from the same species was c.a. 65%. Although the responsibility for the correct molecular identification of species rests on researchers, the errors found in already published sequences data have not been re-evaluated so far. On the basis of the standard sampling technique we have estimated with 95% probability that the chances of finding incorrectly described metazoan sequences in the GenBank depend on the systematic group, and variety from less than 1% (Mollusca and Arthropoda) up to 6.9% (Gastrotricha). Consequently, the increasing popularity of DNA barcoding and metabarcoding analysis may lead to overestimation of species diversity. Finally, the study also discusses the sources of the problems with amplification of non-target sequences.

## Introduction

In 1994 Folmer et al. [1] described “universal” DNA primers: forward LCO1490 and reverse HCO2198 (commonly referred to as the “Folmer primers”), to amplify the most conservative protein-coding gene in metazoan invertebrates—the mitochondrial cytochrome c oxidase subunit I (*cox1*) gene fragment of the length approximately 700 bp. These primers were successfully used in amplifying the *cox1* gene fragment for more than 80 invertebrate species, proving their universality across diverse metazoan taxa [2]. This in turn resulted in the *cox1* gene, due to its robustness and reliability, being accepted as the standard taxon barcode for most animals (e.g., [3]). Moreover, a high rate of substitution in the third codon position of this molecular marker allowed to apply it for phylogeny both at the population and species level [4]. In consequence, Folmer primers appear to be irreplaceable in polymerase chain reaction (PCR) for phylogenetic studies of diverse invertebrate organisms, and they make phylogenetic analyses at the species (and also higher) taxonomic level less complicated. Moreover, “barcoding” COI sequences (barcodeoflife.org) have also been widely used to determine unknown species’ identity, and the records are given the keyword BARCODE in the GenBank. Unknown COI sequences are matched with the sequences deposited in the GenBank, and identification of species is frequently carried out by referring the database for comparison [5]. More recently, the utility of these barcode primers have also been shown in application to metabarcoding analyses (e.g., [6]).

In the period between 1994 and 2016 the number of scientific articles referring to Folmer primers reached nearly 17000 in Google Scholar. Moreover, the citation count for the original Folmer et al. [1] paper in the databases at Web of Science crossed 7600 and this number is constantly increasing. In order to maintain the broad taxonomic utility of COI sequences, a high level of degeneracy in updated Folmer primers is necessary (e.g., [7]). Nonetheless, it has been already shown that in numerous analyses of COI sequences these primers mismatch many metazoans [8–10]. To overcome the limitations of the Folmer primers, alternative primers have been developed [11]. In 2013 the Barcode of Life Database (the BOLDSystems) revealed c.a. 418 different primers targeting COI sequences for various taxa. Besides this, some researchers also applied considerably reduced primer annealing temperatures to resolve these amplification problems [12].

Nevertheless, in the DNA barcode approach, species identification could be carried out without any *a priori* knowledge about the investigated samples. This approach might be used for DNA barcode recovery from mixed environmental probes or identification of problematic specimens with ambiguous morphology—especially when the material is damaged or only parts of the body are present. Thus, in those cases universal primers, e.g. Folmer primers, seem to remain a reasonable solution. However, they can also co-amplify the nuclear mitochondrial pseudogenes—numts or “COI-like” sequences [13, 14] and microorganisms [15]. Finally, it can lead to overestimation of species diversity, as well as global biodiversity patterns [14]. Siddall et al. [15] analysed homologous priming site sequences obtained from prokaryotic and eukaryotic whole genome data, and they indicated the presence of targeted Folmer primer sequences. What is more, they showed that several genomes of bacterial species exhibited more contiguously matched nucleotides for the reverse primer than most animals.

The phenomenon of contaminant amplification of non-target taxa is quite common and mismatched sequences are still published. Using the universal Folmer primers to amplify the *cox1* gene of crustacean fairy shrimp *Branchipus schaefferi* (Fischer 1834) we also obtained bacterial sequences which were described as metazoan in GenBank. This finding encouraged us to examine more closely the cases of mismatched and mislabelled sequences stored in the available on-line databases. In this case we focused on (i) analysis of the bacterial sequences (obtained with the universal Folmer primers) deposited in the GenBank as invertebrate sequences and their influence on DNA barcoding (and metabarcoding) of metazoan species, (ii) estimating the proportion of incorrectly flagged sequences deposited in the GenBank using the random sampling procedure. In addition, we discuss the problems with the amplification of non-target sequences and the possibilities of preventing incorrectly flagged sequences in further analyses.

## Materials and methods

### DNA extraction, PCR amplification, and DNA sequencing

Samples of muscular tissues from 25 specimens of *Branchipus schaefferi* (Crustacea: Anostraca) collected in 2014 from a temporary water body located in a military training ground in western Poland (Poznań-Biedrusko; ca. 52°29'N, 16°51'E) were immediately isolated from the fresh thorax after the sampling. The total DNA was extracted from the samples in accordance with the protocol of the Biotrace Genomic Extraction GPB Mini Kit (GenoPlast) (see also [16]).

Until now, only the 18S rRNA gene has been used in genetic analyses of several specimens of *B*. *schaefferi* [16, 17]. Thus, molecular identification of species was conducted with these barcode sequences in accordance with the procedure described by Mioduchowska et al. [16].

The fragment of mtDNA spanning the *cox1* gene was amplified with the universal primers HCO2198 and LCO1490 [1]. The polymerase chain reactions (PCRs) were performed in a 20 μL volume containing 0.8× JumpStart Taq ReadyMix (1 U of JumpStart Taq DNA polymerase, 4 mM Tris–HCl, 20 mM KCl, 0.6 mM MgCl2, 0.08 mM of dNTP; Sigma-Aldrich, Germany), 0.4 μM of forward and reverse Folmer’s primers, and about 100 ng of DNA. The *cox1* gene fragments were amplified under the following conditions: the initial denaturation at 94°C for 5 min was followed by 30 cycles of 94°C for 30 s, 44°C for 1 min (gradient PCR amplification ranging from 42 up to 52°C was applied to determine the optimal annealing temperatures), and 72°C for 1 min and at the end at 72°C for 5 min. The reactions were conducted in a Biometra TProfessional thermocycler. The products of amplification were separated by 1% agarose gel electrophoresis in a 1x SB buffer and then visualized with ethidium bromide in UV light. The PCR products were purified with alkaline phosphatase and exonuclease I (Thermo Scientific, USA) treatment in accordance with the protocols and sequenced with the BigDye^™^ terminator cycle sequencing method.

The obtained sequences have been deposited in the GenBank under accession numbers given in Table 1.

**Table 1:** The list of correctly and incorrectly flagged sequences of eukaryotic and microbial *cox1* gene fragment applied in our study.

***Branchipus schaefferi* (correct sequence; accession: MF627724 (*Branchipus*_*schaefferi*_haplotype_B1); present study)**					
**Sequences producing significant alignments from first five species**	**Accession**	**Query coverage**	**Identity**	**E value**	**Literature data**
*Branchipus* sp.	KP702848	100%	99%	0.0	Gandolfi et al., unpublished
*Streptocephalus texanus*	KT583299.1	99%	83%	2e-162	Halliburton et al., unpublished
*Branchinella buchananensis*	AF308960.1	99%	83%	4e-163	[40]
*Branchinella frondosa*	AF308958.1	99%	82%	2e-160	[40]
*Simulium verecundum*	KR685968.1	99%	83%	5e-156	[41]
**microbial contaminants *Branchipus schaefferi* from *Aeromonas* sp. (incorrectly flagged sequence; accessions: MF627721 (Aeromonas_A1), MF627722 (Aeromonas_A2), MF627723 (Aeromonas_A3); present study)**					
**Sequences producing significant alignments from first five species**	**Accession**	**Query coverage**	**Identity**	**E value**	**Literature data**
*Aeromonas hydrophila*	CP006579.1	100%	88%	0.0	[42]
*Aeromonas media*	CP007567.1	100%	87%	0.0	[43]
*Aeromonas schubertii*	CP013067.1	100%	82%	3e-153	[44]
*Ferrimonas balearica*	CP002209.1	100%	80%	8e-141	[45]
*Lacimicrobium alkaliphilum*	CP013650.1	100%	80%	8e-141	Kim and Lee, unpublished
***Patelloida striata* (correct sequence; accession: AB238524.1; [25])**					
**Sequences producing significant alignments from first five species**	**Accession**	**Query coverage**	**Identity**	**E value**	**Literature data**
*Patelloida* sp.	AB238526.1	99%	80%	6e-136	[25]
*Patelloida ryukyuensis*	KM221059.1	99%	74%	7e-91	[46]
*Patelloida pygmaea*	AB161601.1	99%	74%	1e-88	[23]
*Patelloida heroldi*	AB161622.1	99%	74%	4e-88	[23]
*Patelloida conulus*	AB161608.1	99%	73%	5e-87	[23]
***Patelloida striata* (incorrectly flagged sequence; accession: AB161589.1; [23]) and *Tetranchyroderma* sp. 3 (incorrectly flagged sequence; accession: JF432035.1; [24])**					
**Sequences producing significant alignments from first five species**	**Accession**	**Query coverage**	**Identity**	**E value**	**Literature data**
*Tetranchyroderma* sp.	JF432035.1	100%	98%	0.0	[24]
*Endozoicomonas montiporae*	CP013251.1	100%	83%	1e-158	[47]
*Pseudomonas* sp.	AP014637.1	100%	78%	4e-138	[48]
*Marinobacter adhaerens*	CP001978.1	98%	79%	7e-123	[49]
*Hahella chejuensis*	CP000155.1	100%	79%	1e-125	[50]
***Tetranchyroderma pachysomum* (correct sequence; accession: JF432029.1; [24])**					
**Sequences producing significant alignments from first five species**	**Accession**	**Query coverage**	**Identity**	**E value**	**Literature data**
*Tetranchyroderma cirrophorum*	JF432028.1	99%	79%	1e-132	[24]
*Tetranchyroderma thysanophorum*	JF432025.1	98%	78%	1e-118	[24]
*Tetranchyroderma hirtum*	JF432023.1	89%	76%	4e-100	[24]
*Oregodasys tentaculatus*	JF432021.1	98%	74%	2e-79	[24]
*Pseudostomella etrusca*	JF432026.1	97%	73%	3e-83	[24]

### DNA sequence analysis and phylogenetic tree reconstruction

The obtained sequences were aligned manually using BioEdit 5.0.9 [18] and consensus sequences were created. The alignments were prepared in ClustalX 1.81 [19] with the default settings for the COI marker. The BLAST application (Basic Local Alignment Search Tool; [20]) was used for the homology search and to browse the sequences deposited in the NCBI database. To check the indels and internal stop codons, the sequences of the *cox1* gene fragments were translated into amino acid sequences with the EMBOSS-TRANSEQ application [21].

The phylogenetic analyses of the obtained sequences, which closely matched the query sequences downloaded from the GenBank, were carried out with the MEGA software package (version 6.06; [22]). Some of these sequences were found to be described in the GenBank as belonging to taxa that are phylogenetically distant from Crustacea, mislabelled as marine gastropod *Patelloida striata* (accession: AB161589.1; [23]) and the marine gastrotrich *Tetranchyroderma* sp. 3 [24]. Thus, we performed another search for other sequences with similar descriptions (or closely related) taxa, and we found entries for *Patelloida striata* (accession: AB238524.1; [25]) and *Tetranchyroderma pachysomum* [24]. We included them together with the closely matched query sequences in the phylogenetic reconstruction tree. The genetic distances were calculated with the MEGA software package (version 6.06; [22]) as p-distance values of Nei & Kumar [26]. The evolutionary model was established with jModelTest 2.1.4 [27] with the assumptions of the Akaike Information Criterion (AIC) [28]. The General Time Reversible (GTR) model with the gamma-distributed rate parameter was chosen as the best-fit and the Maximum Likelihood (ML) method was also applied. For the assessment of the node support 1000 bootstrap iterations were performed.

In the current paper we use the term “correct sequences” for sequences obtained from target species and “incorrect sequences” for those acquired from non-target species.

### Testing the probability of detecting incorrectly flagged sequences deposited in the GenBank

We checked the NCBI nucleotide database for all COI sequences of Gastrotricha, Mollusca, and Arthropoda to estimate the percentage of incorrectly identified sequences. These analyses were performed for taxonomic groups in which sequence errors have been already observed. Since there is no obvious consensus on the metadata definition and gene vocabulary in the NCBI database (e.g. some of the deposited sequences had only “cytochrome c oxidase subunit I” in definition part of metadata, other entries had CO1 acronym instead of COI), we used following query with proper conditions to obtain as many as possible sequences related only to cytochrome c oxidase subunit I:

′(<taxonname>[Organism])AND("oxidasesubunitI"OR"oxidasesubunit1"OR"CO1"OR"COI"))NOT("subunitII"OR"subunit2"OR"subunitIII"OR"subunit3")′

where for <taxon name> we substituted: Gastrotricha, Mollusca or Arthropoda. According to the binomial distribution, the probability of detecting at least one misidentified record in a sample is given by:

$$\text{P}\left(\text{X}>0\right)=1-{\left(1-\text{p}\right)}^{\text{n}}$$

where P is the probability of detection, p is the proportion of misidentified records in a population, and n is the sample size (e.g., [29]). Thus, we put forward a hypothesis that at least 1% of sequences deposited for each investigated taxa are misidentified. To calculate the sample size the above equation needs to be transformed into:

n=log(1−P(X>1))/log(1−p)

Using 0.95 as the probability of detection and 0.01 as the ratio of misidentified sequences in the population, the calculated sample size equals 300. In other words, there is a 95% chance of finding at least 1 misidentified sequence in a sample of size 300, assuming that the true ratio of misidentified sequences in the whole population is 0.01. Taking these calculations into account, we decided to randomly sample 300 sequences for each taxon. In the case of Gastrotricha the total number of IDs was 363, and hence we decided to use all of them in the analyses to calculate the parameter for this population. Then, we downloaded all sequences related to the sampled IDs. This procedure was performed in R 3.4.1 [30] with package rentrez 1.1.0 [31]. For the obtained results we calculated 95% confidence intervals using the exact method with R package binom 1.1–1 [32]. The comparison with the GenBank records was carried out with BLAST to identify the sequences that were homologous to the obtained *cox1* gene fragment.

Additionally, a stochastic calculation was performed to estimate the 5th, 50th, and 95th percentile of the potential number of misidentified sequences in the database (absolute values). It was calculated by multiplying 10000 random probabilities from the beta distribution Beta (α, β) by the number of all obtained sequences for Arthropods and Molluscs separately. The shapes of the beta distribution were calculated as follows: α = k +1 and β = n − k + 1, where k is the number of positive results and n is the sample size. We have also performed a simple stochastic simulation of the number of erroneous sequences that might be sampled from the GenBank database for Arthropods and Molluscs with 3 different sample sizes: 300, 1000 and 10000. To perform this calculations we ran 10000 iterations over binomial distributions B (n, p), where n is the sample size and p is the probability from the previous Beta (α, β) iterations. We have presented the 5th, 50th and 95th percentile for all results. The above calculations were performed in R 3.4.1 [30].

## Results

The examined specimens were classified as *Branchipus schaefferi* on the basis of morphology as well as 18S rRNA sequences (GenBank accession number: KU645889). The Folmer primers used in the analysis amplified not only *cox1* of *B*. *schaefferi* but also of bacterial origin. There were no differences in the size and quality of the obtained PCR products. Despite the high quantity and quality of the PCR products visualized on agarose gel, the sequencing results were poor due to the presence of mixed amplicons. Only 7 out of 25 analysed samples were successfully sequenced, while the other sequence chromatograms showed the effect of heteroplasmy [33]. Finally, we obtained two COI sequences (one haplotype) of *B*. *schaefferi* and five non-crustacean sequences (three haplotypes). These were further analysed as to the expected matches and nearest neighbours (BLAST searches), and the analysis has revealed non-target amplification of microbial contaminants from *Aeromonas* sp. No stop codons and indels have been observed for these seven sequences, and the translation was successfully carried out with -2^nd^ reading frame. We have also managed to overcome the problems with the co-amplification by designing species-specific primers for weak sequences derived from mixed products. In this way we have obtained only target species—the COI sequences of *B*. *schaefferi*–and we have acquired non-target species with another pair of species-specific primers—the COI of *Aeromonas* sp. (Mioduchowska et al., in preparation).

**Fig 1:**
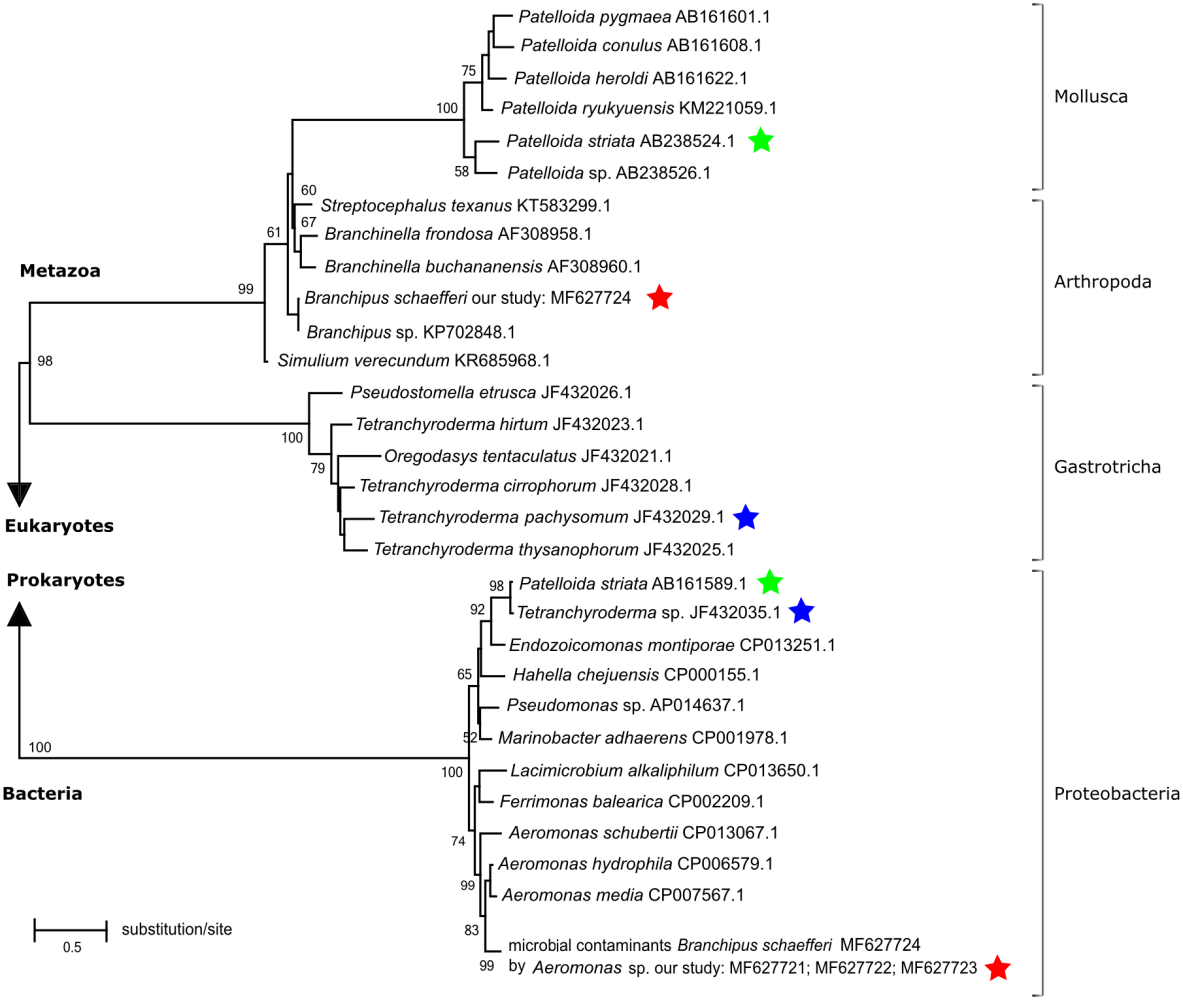
Maximum Likelihood tree showing evolutionary relatedness of eukaryotic and prokaryotic COI barcoding sequences. The bootstrap values are shown at the nodes. The used colors of asterisk indicate both correctly and incorrectly described sequences for the same species. In the case of the sequences obtained in our study for *B*. *schaefferi* the sequences were correctly labeled and put into the GenBank. More information about the applied sequences is provided in Table 1.

We also reconstructed the phylogenetic relationships between our sequences and the nearest neighbours deposited in the GenBank. The Maximum Likelihood analyses (Fig 1) placed the obtained sequences among general clades of Metazoa (Arthropoda above all) and bacteria (all of them were Proteobacteria). Performed bootstrap analysis shows high support values: 100% support for a Eukaryota/Prokaryota bipartition, 98% support for Metazoa genomic COI, and 100% support for bacteria.

Surprisingly, two sequences of the nearest neighbours classified in the analysis as bacterial clades had labels of clearly metazoan species in the GeneBank database. These sequences were described as gastropod *Patelloida striata* ([23]; AB161589.1, BOLD: ACY9706) and gastrotrichan *Tetrachyroderma* sp. ([24]; JF432035.1). However, other sequences assigned to these species are also present in the GeneBank, and these in turn match the Metazoa clade, and they have been placed on the phylogenetic tree in positions fairly fitting to their phylogenetic relationships. These were one sequence of *P*. *striata* ([25]; AB238524.1, BOLD: AAI4206) and one sequence of *Tetrachyroderma pachysomum* ([24]; JF432029.1) (Fig 1).

The average genetic distance (p-distance) between the COI sequences of Metazoa species and the analogous COI fragment derived from whole-genome sequencing of bacteria was 65%. The mean p-distance values between the sequences in the clades ranged between 2% and 67%–the values for the Metazoan clade and between 2% and 27%–values for the bacteria clade. Furthermore, the genetic distance between the incorrect and correct sequences of the same species was as follows: *Branchipus schaefferi*– 62%, *Patelloida striata*– 64%, and *Tetrachyroderma* sp.– 68%.

According to our query, the number of all Mollusca and Arthropoda COI sequences deposited in the GenBank was 109502 and 2008162 respectively. In the case of Gastrotricha we analysed all 363 sequences deposited in the GenBank. In our random sample of sequences, we have observed errors in Gastrotricha and Mollusca. However, these errors not always were related to the description of the metazoan sequence as a bacterium, but some mistakes have been observed also among the metazoan species. Among the sequences of Mollusca we identified one incorrectly flagged sequence, which had been classified as a metazoan sequence (GenBank accession number: JN100021.1; deposed in 2011 by Valenzuela and co-authors; there is no information indicating exactly which primers were used). In the case of Gastrotricha two sequences were of bacterial origin (GenBank accession numbers: JF432035.1, JF432024.1; [24]; Folmer primers were used), one was of fungal origin (GenBank accession number: KP878838.1; deposited in 2015 by Atherton; Uro3HF and Uro3HR primers were used), and 22 sequences came from other metazoan species (nonetheless, some of the incorrectly flagged sequences could be numts, see supplementary data S1 Table; [24, 34–39]).

On the basis of our analyses we can conclude with 95% probability that less than 1% of the sequences among Arthropoda and 1% of Mollusca sequences are erroneously classified. When expressed in terms of 95% confidence intervals, the ratio of misidentified sequences in Arthropoda is close to 0, with lower and upper boundary 0 and 0.012 respectively, while in Mollusca the ratio is 0.003 with lower and upper boundary > 0.001 and 0.018 respectively. In other words, approximately 4601 (5th percentile: 348, 95th: 19890) of the erroneous sequences are deposited in the Arthropoda group and 615 (5th percentile: 132 and 95th: 1673) of the erroneous sequences are deposited for Mollusca. The results of the simulation show that when we sampled 300, 1000 or 10000 sequences deposited in the GenBank for Arthropoda, we found 1 (0, 4), 2 (0, 11) or 24 (1, 101) erroneous sequences, respectively. For Molluscs the values were 1 (0, 6), 5 (0, 17), 56 (11, 154)–for sample size 300, 1000 and 10000, respectively (5th and 95th percentile values in brackets). There are also 25 incorrect sequences deposited in the GenBank for Gastrotricha, roughly 0.6% of the sequences belong to bacteria, 0.3% to fungi, and surprisingly, 6.06% of them belong to other metazoan species and probably to numts. The incorrect sequences represent approximately 6.9% of all deposited sequences in this group of organisms.

The sequences presented in Table 1 that were described as correct (originating from the target species), as well as those incorrect (originating from the non-target species) were amplified with the Folmer primers. Similarly, almost all amplifications of the incorrect Gastrotricha that are included in S1 Table were carried out with these universal primers. However, there is no information about which primers were used to amplify the *cox1* gene fragment of the incorrectly flagged four *Urodasys poculostylis* sequences (Atherton 2015, unpublished; S1 Table). Finally, newly designed internal primers, combined with the modified Folmer primers, were used for one *cox1* gene sequence of *Mesodasys* sp. ([36]; S1 Table).

## Discussion

The metazoan DNA barcoding with the Folmer primers enables quick and easy species identification, detection and discovery. However, some authors (e.g., [51–53]) have showed that the Folmer primers, one of the most frequently used research tools for COI amplification, are not so “universal” for some taxa, though at the same time they seem too “universal” for non-target bacterial genomes (e.g., [15]). Moreover, these primers are poorly conserved across some metazoan representatives, e.g. gastropods [8], nematodes [9, 54], and echinoderms [10].

The universality of the Folmer DNA primers was discussed by Sharma and Kobayashi [55], who showed some weaknesses of this method. First of all, the limitations in the universality are related to the original design procedure based on three sense and six anti-sense strands and comparison of the *cox1* gene conserved regions across the 15 taxa. During the analysis of 130,843 variations in the primer region of COI sequences from the kingdom Animalia they observed that the reverse primer was truly conserved, but the forward primer contained only four conserved regions and approximately 50% conserved regions for vertebrates, fungi, and ascidians. The problems associated with one or both Folmer primers (especially the high nucleotide variability in the forward primer) sometimes cause a relatively low amplification rate reported for certain taxonomic groups. This has led to designing species-specific primers (e.g., [56]).

The correct species identification based on DNA barcoding might also be hindered due to numt co-amplification. Moulton et al. [57] showed that even increased primer specificity was not sufficient to eliminate completely numt co-amplification, which was contradictory to the suggestions of Song et al. [14]. Moreover, Moulton et al. [57] showed that many numts do not have indels and stop codons, which is a serious problem for DNA barcoding. In addition, these findings make it more difficult to distinguish numt sequences from mitochondrial orthologues. Furthermore, Siddall et al. [15] compared homologous priming site sequences obtained from complete prokaryotic genome data and mitochondrial eukaryotic genome data, and they showed that the first 10 nucleotide positions of the forward Folmer primer demonstrated variability across a wide range of eukaryotes and prokaryotes. The reverse Folmer primer showed variation only in the third codon positions and all eukaryotic nucleotide variants were also present among the prokaryotes. Furthermore, some gamma-proteobacteria exhibited more contiguously matched nucleotides for the reverse Folmer primer than from the metazoan taxa.

We have also observed amplification and sequencing of non-target bacterial templates in our analysis. We found that the bacterial sequences amplified from *Branchipus schaefferi* were in 88% identical (query coverage was 100% and E-value was 0.0) to *Aeromonas* sp. sequences. The source of the bacterial DNA in our samples could derived from the gut contents or the surface of the thorax tissues (see also [7]). Nonetheless, this remains a speculation and more detailed research, such as metagenomic analysis is required. We were very careful during the sampling of *B*. *schaefferi* tissues and we applied laboratory procedures to minimize anomalous results and to exclude bacterial contamination or mixed templates (e.g., laboratory errors). However, anaerobic *Aeromonas* occur quite frequently and autochthonously in aquatic environments [58]. Besides this, *Aeromonas* sp. is known to be a fish pathogen [59] and commonly occur in the Medicinal leech *Hirudo medicinalis* in symbiosis [60, 61] or in nematodes for which it is toxic [62].

It is intriguing that bacterial COI sequences were found also in other metazoa species, and they have been erroneously deposited in a public database like the GenBank, e.g. as *Patelloida striata* [23] and *Tetranchyroderma* sp.3 [24]. Interestingly, the same authors described both correctly [24, 25] and incorrectly flagged sequences [23, 24] but they were not aware of the fact that those sequences had been erroneously labelled. Unfortunately, we cannot say anything about the potential sources of the bacterial DNA in these samples. However, in the case of marine gastrotrich *Tetranchyroderma* sp., as well as the marine limpet mollusc *Patelloida striata*, it is not surprising that bacterial sequences from these species match to the marine gamma-proteobacteria clade. The prokaryotic COI sequences were in 83% identical (query coverage was 100% and E-value was 1e-158) to those of *Endozoicomonas montipore*—aerobic bacterium isolated from the encrusting pore coral *Montipora aequituberculata* collected from seawater [47, 63]. Species of *Endozoicomonas* were frequently found also in various marine invertebrates, e.g., the sea slug [64], sponge [65], and the comb pen shell [66]. Due to the fact that *Endozoicomonas* have positive association with healthy individuals (especially in corals), these bacteria are considered as an essential affiliate of the host holobiont [47].

In most cases not only the primer sequences but also the annealing temperatures typically used for DNA barcoding are crucial for the successful amplification of the target taxon. Given the fact that the Folmer primers are such a poor match to many metazoan species, associating surface bacteria as well as gut bacteria, could be also preferentially amplified under low annealing temperatures [15]. Consequently, too low annealing temperatures are the main reason of contaminant bacterial COI sequence amplification. In the case of our *B*. *schaefferi* sequencing we applied an annealing profile of 44°C that preferentially amplified *Aeromonas* species. As a result, the highest annealing temperature used in the analysis generated weak or no PCR products. Many barcoding sequences deposited in the GenBank have in fact incorrect metazoan labels, as they are products of bacterial contaminants (e.g., [15]). In the case of the correct COI sequences of *Patelloida striata* and *Tetranchyroderma pachysomum* the amplifications were carried out with an annealing profile of 45–50°C and 46°C, respectively [24, 25]. Notwithstanding, the incorrectly flagged COI sequences of these metazoan species were amplified with an annealing profile as follows: 54°C for *Patelloida striata* and 46°C for *Tetranchyroderma* sp. [23, 24]. The obtained results are consistent with the observations of Siddall et al. [15], who claim that stringent annealing temperatures should not be expected to remediate the problem of bacterial contaminant amplification of COI sequences, and poor primer complementarity to metazoan genomes necessitates liberal amplification regimes.

According to Kress & Erickson [67] there are several traits that molecular markers should have to serve as efficient DNA barcode sequences, i.e., they should correctly discriminate the examined species and they should show a range of taxonomic diversity and the presence of universal primer pairs for PCR amplification. Moreover, the relative rate of evolution of mitochondria, as well as nuclear genes, which vary in different organisms, should be also considered [68]. In addition to COI barcode sequences, some other potential barcoding markers exist for various organisms, e.g., the 18S rRNA gene (e.g., [69]). Flanking regions of rRNA gene sequences can be used for universal primer amplification due to their high conservation. Moreover, the 18S rRNA gene is widely regarded as the primary candidate for reconstruction of metazoan relationships [70]. Recently, it has also been shown that this conservative molecular marker is a suitable tool for molecular identification of *B*. *schaefferi* [16].

At present, DNA-based community analyses have offered some alternatives to the traditional methods of species identification, and metabarcoding is one of the most promising techniques to understand taxon diversity. This method enables species detection from a bulk of samples, e.g., environmental samples, gut or faecal contents [36], and it might be a good solution to the problems with proper species identification in the case of cryptic species (e.g., [71]) or poor species-specificity of cyst morphology and biometry, which is used when no active specimens are present (e.g., [72]). Metabarcoding is based on PCR amplification followed by deep sequencing of homologous gene regions. The resulting barcode sequences are then compared to homologous sequences deposited in databases (e.g. the GenBank, BOLDSystems, or created by research groups with special reference libraries) for multiple species identification in complex samples. However, due to the fact that many sequences have been incorrectly annotated in the reference libraries, this method is also prone to generate errors in species identification. Erroneous data that may otherwise potentially lead to misidentification of species should be removed following the first essential step (after obtaining NGS data), where sequence errors can be removed during quality filtering. Nevertheless, species misidentification can also occur during the next step, when the clustering method is applied into operational taxonomic units (OTUs), if only the obtained sequences match the incorrectly described references deposited in the DNA barcodes library (which, as we showed in this study, can be one of the problems with the use of the GenBank database) [73].

It seems that using BLAST search engine to compare homologous sequences deposited in the GenBank is sufficient for correct species identification at the molecular level. Nevertheless, there is a need to develop new algorithms and procedures to find the incorrectly labelled sequences in a more efficient way. For example, comparison of p-distance values calculated between new sequences and the match entries in the GenBank database should be helpful to filter out non-target bacterial genomes contaminants. In our study the values of the p-distance between the correct and incorrect sequences was ca. 65%, and the mean range of such values for the species should be ca. 2% (see also supplementary data S1 Table).

Although the ratio of the metazoan incorrectly flagged sequences annotated in the GenBank depends on the systematic group and varies between less than 1% and 6.96%, the probability of duplicating these errors in barcoding and metabarcoding analyses is high. Due to the small size of some metazoans, e.g. Gastrotricha, and the presence of microsporidian parasites [74] and unknown type of interaction between these organisms and protists [75], we expected that more incorrectly flagged sequences would be observed within these taxonomic groups. The results of the present study confirm our preliminary assumptions. Nevertheless, although we have not found bacterial sequences in Mollusca and Arthropoda, it has been shown that some mistakes are occurring also in these groups (this study, [15]). The ratio of detecting incorrectly described sequences of Gastrotricha originating from other metazoan taxa or probably numts was very high (6.06%). Thus, this is a serious drawback and therefore molecular taxonomy of these groups should be verified by specialists in the field with more attention.

The main problem which arises when researchers obtain a good quality sequence is that it can mislead to believe that it comes from the target species. Moreover, verification of the correctly flagged sequences with the BLAST can be difficult in the case of a new species, for which there are no reference sequences in the available databases. One should also remember that there is only one sequence of the *cox1* deposited in NCBI databases for most of the Gastrotricha species described in S1 Table (10 out of 17 species), which is another difficulty that one can encounter in verification of taxonomic identification. We have identified the incorrectly described Gastrotrich sequences that come from other metazoan species. We consider these sequences to be true mitochondrial sequences due to the translation capacity and lack of gaps. Some of these sequences were highly homologous to the orthologous mtDNA sequence of *Archaphanostoma ylvae* and *Palaemon floridanus* with 99–100% query coverage in the BLAST search results, and the E value from 2e-167 to 0.0, and values of p-distance ranging between 0.000 and 0.220 (S1 Table, No 16–22).

The analysis has also revealed that some incorrectly flagged Gastrotricha sequences with a significant match to non-target metazoan species were marked as “cytochrome c oxidase subunit I like (COI) gene” (S1 Table, No 4, 5, 7–9); some sequences contained internal stop codons, disrupting the open reading frame of the amino acid sequence (S1 Table, No 5, 7, 9), and there were also sequences with gaps (S1 Table, No 1–15). Thus, it seems that the these sequences probably come from nuclear genes (numts). Similarly, Todalo et al. [24] described another c oxidase subunit I-like gene of Gastrotricha (accession: JF432020, JF432021, JF432022, JF432025, JF432034). These sequences do not conform to the general invertebrate mitochondrial sequence pattern, as well as the genetic code of other organisms deposited in the available databases; the sequences with gaps and without translation to their corresponding amino acid sequence. The authors also suggest that the ostensible COI sequences could be numts or one or more modifications of the mitochondrial genetic code within Gastrotricha. Although numts are highly divergent from true mitochondrial sequences, these sequences were significantly similar to other Gastrotricha sequences found in the NCBI database. Hence, it might seem that they come from the target species, and this approach is also a major problem.

The lack of a unified coding system for the already identified sequences makes finding the relevant data difficult. During the procedure of finding the proper query statement for the NCBI database we managed to find many sequences related to one gene (*cox1*) with a few different formats of description (e.g. "COI" vs. "CO1", "cytochrome c oxidase" vs. "cytochrome oxidase"). This lack of uniformity and consistency results in difficulties in formulating the right query statement which would give the desired results. Therefore, we postulate that there is still an urgent need of unifying the description format of the sequences deposited in the GenBank.

There is a number of databases which are useful in the Linnaean system of animal taxonomy (e.g. www.fauna-eu.org; www.catalogueoflife.org). The Barcode of Life Data System (http://www.boldsystems.org) is currently the most popular integrated bioinformatic platform, which supports the analytical pathway ranging from specimen collection to tightly validated barcode library. It contains almost 6,000,000 barcodes, providing a direct link between the specimen data, digital images, taxonomy and assigning sequences to the BINs (Barcode Index Numbers) (e.g., [76, 77]). Furthermore, the BOLD data analysis tools enable screening the submitted sequences for some common contaminants and endosymbionts, such as *Wolbachia*, and flagging them as contaminants. However, it is quite striking that incorrect sequences were also found in the BOLD database. For example, there are both correct [25] and incorrect sequences [23] for *Patelloida striata* in this database. This raises a question whether it is possible to reduce considerably the number of such mistakes in databases even with strict measures and procedures. Nevertheless, the barcoding method should not be impugned, but a more careful approach is needed in those initiatives. There is no doubt that this will be the most valuable approach in identification of species in the future.

We are also determined to continue the research into the identification of incorrectly described sequences. A good solution to the problems associated with errors in species identification could be an on-line application, which would be based on integrative taxonomy (a combination of both morphology and DNA data). A preliminary concept of integrative taxonomy as a combination of both morphology and DNA data in an on-line application has been already proposed by Kur et al. [78], and similar research is in progress in our laboratories. A similar approach in developing a standardized on-line data repository has been adopted in the case of tardigrade taxonomy (www.tardigrada.net/register, [79]). The digitization of morphological keys will be a novelty in our application when compared to the BOLD system (which despite numerous advantages is not based on the integrated taxonomy, but it is only an integrated bioinformatic tool, compare e.g., [80]). The application will allow species identification at the morphological level on the basis of a diagnosis of a number of morphological features. Moreover, the integration of comparative morphological and molecular taxonomy will also allow precise species identification and delimitation. We are convinced that the conceptual multi-platform web application, based on a combination of molecular and morphological data, will be available for both specialists in this field of research and broad public.

## Conclusions

Although the responsibility for the right molecular identification of species rests upon researchers, there is no doubt that many sequences deposited in the GenBank should be verified more thoroughly. This fact should be seriously taken into consideration especially by the researchers who use universal primers, e.g. Folmer primers, which have a high specificity to bacterial genome. As a consequence, some of the accounts of new species described only at the molecular level remain questionable. Moreover, this diagnostic method of species identification can also lead to overestimation of species number, which in turn can result in numerous mistakes. Therefore, there is an urgent need to review and correct the available data to prevent similar errors in further research. The present study clearly shows that the best solution to eliminate incorrect sequence flagging is to perform more thorough comparative analyses of homologous sequences deposited in the available databases, both the NCBI and BOLD.

## Supporting information

S1 Table The list of incorrectly flagged *cox1* gene fragment sequences of Gastrotricha originated from another metazoan species. (DOCX)
