# Supplementary material for: Instances of erroneous DNA barcoding of metazoan invertebrates: Are universal cox1 gene primers too “universal”?
Source: PLoS One. 2018 Jun 22;13(6):e0199609. doi: 10.1371/journal.pone.0199609 (PMC6014667; doi:10.1371/journal.pone.0199609)
Supplement: S1 Table — (DOCX) [file pone.0199609.s001.docx]

**S1 Table. The list of incorrectly flagged *cox1* gene fragment sequences of Gastrotricha originated from another metazoan species.**

| **No.** | **Gastrotricha**  **Accession** | **Literature data** | **Sequences producing significant alignments from one of the most similar species (Phylum)**  **Accession** | **Max score** | **Total score** | **Query coverage** | **E value** | **Identity** | **Gaps** | **Average uncorrected**  **p-distances***  **(base pair fragment of COI sequences applied**  **to analysis)** |
| --- | --- | --- | --- | --- | --- | --- | --- | --- | --- | --- |
| 1 | *Diuronotus aspetos*  KX531007.1 | [38] | *Angaria delphinus* (Mollusca)  KX298893.1 | 843 | 1362 | 96% | 0.0 | 75% | 14/1318 (1%) | 0.267 (691) |
| 2 | *Mesodasys* sp.  KC706821.1 | [36] | *Diglyphus isaea* (Arthropoda)  DQ149178.1 | 201 | 201 | 87% | 8e-48 | 80% | 6/228  (2%) | 0.543 (222) |
| 3 | *Redudasys* sp.  KJ950123.1 | [37] | *Nymphes myrmeleonoides* (Arthropoda)  KJ461322.1 | 354 | 354 | 98% | 2e-93 | 73% | 19/652  (2%) | 0.197 (648) |
| 4 | *Neodasys chaetonotoideus*  JQ798675.1  *** | [35] | *Coelotes* sp. (Arthropoda)  KY779021.1 | 336 | 336 | 84% | 4e-88 | 74% | 6/558  (1%) | 0.313 (555) |
| 5 | *Neodasys uchidai*  (UNVERIFIED)  JQ798676.1  **, *** | [35] | *Coelotes lamellatus* (Arthropoda)  KY779016.1 | 253 | 253 | 85% | 4e-63 | 71% | 7/569  (1%) | 0.605 (553) |
| 6 | *Xenotrichula* sp.  JN185545.1 | [34] | *Astata* sp.  (Arthropoda)  KR877981.1 | 365 | 365 | 85% | 8e-97 | 76% | 19/563  (3%) | 0.602 (544) |
| 7 | *Ptychostomella* sp.  JF432033.1  **, *** | [24] | *Scolia oculata* (Arthropoda)  AB851898.1 | 221 | 221 | 83% | 2e-53 | 70% | 20/555  (3%) | 0.914 (549) |
| 8 | *Paraturbanella pallida*  JF432045.1  *** | [24] | *Basilia truncata* (Arthropoda)  AB632537.1 | 358 | 358 | 93% | 1e-94 | 72% | 55/660  (8%) | 0.460 (613) |
| 9 | *Ptychostomella tyrrhenica*  JF432027.1  **, *** | [24] | *Lychnocolax* sp. (Arthropoda)  JN082793.1 | 127 | 127 | 71% | 4e-25 | 67% | 37/484 (7%) | 0.549 (450) |
| 10 | *Macrodasys* sp.  JF432052.1 | [24] | *Amaurobius borealis* (Arthropoda)  KM839332.1 | 390 | 390 | 99% | 2e-104 | 73% | 8/660  (1%) | 0.330 (654) |
| 11 | *Turbanella lutheri*  JF432051.1 | [24] | *Alysiinae* sp. (Arthropoda)  KR782533.1 | 376 | 376 | 99% | 5e-100 | 74% | 33/669 (4%) | 0.482 (651) |
| 12 | *Xenotrichula intermedia*  JF432048.1 | [24] | *Longitarsus anchusae* (Arthropoda)  KM445196.1 | 315 | 315 | 98% | 1e-81 | 71% | 16/658 (2%) | 0.357 (650) |
| 13 | *Mesodasys littoralis*  JF432044.1 | [24] | *Orientzomus* sp. (Arthropoda)  KY573364.1 | 288 | 288 | 83% | 2e-73 | 73% | 31/559 (5%) | 0.539 (511) |
| 14 | *Mesodasys laticaudatus*  JF432043.1 | [24] | *Euconnus maeklinii* (Arthropoda)  KJ963315.1 | 315 | 315 | 98% | 1e-81 | 71% | 6/648  (0%) | 0.360 (645) |
| 15 | *Lepidodasys unicarenatus*  JF432039.1 | [24] | *Saphonecrus connatus* (Arthropoda)  EF486878.1 | 304 | 304 | 91% | 2e-78 | 72% | 10/606  (1%) | 0.351 (601) |
| 16 | *Tetranchyroderma papii*  JF432030.1 | [24] | *Archaphanostoma ylvae* (Xenacoelomorpha)  KM527298.1 | 600 | 600 | 99% | 2e-167 | 80% | 0/657  (0%) | 0.220 (657) |
| 17 | *Urodasys poculostylis*  KP878835.1 | Atherton, 2015, unpublished | *Palaemon floridanus* (Arthropoda)  KC019186.1 | 1180 | 1180 | 100% | 0.0 | 99% | 0/657  (0%) | 0.002 (657) |
| 18 | *Urodasys poculostylis*  KP878836.1 | Atherton, 2015, unpublished | *Palaemon floridanus* (Arthropoda)  KC019186.1 | 1186 | 1186 | 100% | 0.0 | 100% | 0/657  (0%) | 0.000 (657) |
| 19 | *Urodasys cf. poculostylis*  KP878852.1 | Atherton, 2015, unpublished | *Palaemon floridanus* (Arthropoda)  KC019186.1 | 1178 | 1178 | 100% | 0.0 | 99% | 0/657  (0%) | 0.003 (657) |
| 20 | *Urodasys cf. poculostylis*  KP878854.1 | Atherton, 2015, unpublished | *Palaemon floridanus* (Arthropoda)  KC019186.1 | 1097 | 1097 | 100% | 0.0 | 99% | 0/618  (0%) | 0.006 (618) |
| 21 | *Urodasys cf. poculostylis*  KP878855.1 | Atherton, 2015, unpublished | *Palaemon floridanus* (Arthropoda)  KC019177.1 | 1076 | 1076 | 100% | 0.0 | 99% | 0/618  (0%) | 0.015 (618) |
| 22 | *Urodasys poculostylis*  KP878853.1 | Atherton, 2015, unpublished | *Palaemon floridanus* (Arthropoda)  KC019177.1 | 1034 | 1034 | 100% | 0.0 | 100% | 0/573  (0%) | 0.000 (573) |

*****average uncorrected genetic p-distances between some species of Gastrotricha values from 0.255 to 0.382 and within the species –
from 0.007 to 0.081 (see [39])

** internal stop codons

*** cytochrome c oxidase subunit I – like gene
